# Global fund financing to the 34 malaria-eliminating countries under the new funding model 2014–2017: an analysis of national allocations and regional grants

**DOI:** 10.1186/s12936-016-1171-3

**Published:** 2016-02-25

**Authors:** Brittany Zelman, Melissa Melgar, Erika Larson, Allison Phillips, Rima Shretta

**Affiliations:** The Global Health Group, University of California, San Francisco, San Francisco, CA USA

**Keywords:** Global fund to fight AIDS, Tuberculosis and malaria, Malaria elimination, Financing, New funding model, Regional grant

## Abstract

**Background:**

The Global Fund to Fight AIDS, Tuberculosis, and Malaria (GFATM) has been the largest financial supporter of malaria since 2002. In 2011, the GFATM transitioned to a new funding model (NFM), which prioritizes grants to high burden, lower income countries. This shift raises concerns that some low endemic countries, dependent on GFATM financing to achieve their malaria elimination goals, would receive less funding under the NFM. This study aims to understand the projected increase or decrease in national and regional funding from the GFATM’s NFM to the 34 malaria-eliminating countries.

**Methods:**

Average annual disbursements under the old funding model were compared to average annual national allocations for all eligible 34 malaria-eliminating countries for the period of 2014–2017. Regional grant funding to countries that are due to receive additional support was then included in the comparison and analysed. Estimated funding ranges for the countries under the NFM were calculated using the proposed national allocation plus the possible adjustments and additional funding. Finally, the minimum and maximum funding estimates were compared to average annual disbursements under the old funding model.

**Results:**

A cumulative 31 % decrease in national financing from the GFATM is expected for the countries included in this analysis. Regional grants augment funding for almost half of the eliminating countries, and increase the cumulative percent change in GTFAM funding to 32 %, though proposed activities may not be funded directly through national malaria programmes. However, if countries receive the maximum possible funding, 46 % of the countries included in this analysis would receive less than they received under the previous funding model.

**Conclusions:**

Many malaria-eliminating countries have projected national declines in funding from the GFATM under the NFM. While regional grants enhance funding for eliminating countries, they may not be able to fill country-level funding gaps for local commodities and implementation. If the GFATM is able to nuance its allocation methodology to mitigate drastic funding declines for malaria investments in low transmission countries, the GFATM can ensure previous investments are not lost. By aligning with WHO’s Global Technical Strategy for Malaria and investing in both high- and low-endemic countries, the Global Fund can tip the scale on a global health threat and contribute toward the goal of eventual malaria eradication.

## Background

Of the approximate 100 countries with endemic malaria, 34 were defined in 2010 as malaria-eliminating (see Table 1), defined here as a country that has a national or subnational evidence-based elimination goal and/or is actively pursuing elimination (zero malaria transmission) within its borders [1]. Among these 34 countries, 78 % of financing for malaria programmes has been provided by governments themselves [2]; however, the percentage of domestic funding can vary widely from country to country, ranging from under 10 % in some low- and lower–middle-income countries (LMICs) such as the Philippines and Tajikistan, and up to 100 % in upper–middle to high-income countries such Costa Rica, South Korea, and Turkey [3].

**Table 1:** 34 malaria-eliminating countries, national elimination goals (as of 2015), and study inclusion status

Country	National elimination goal	Eligible for national funding in 2014	Eligible for funding through a regional initiative	Meets inclusion criteria for this analysis?
Eastern Mediterranean and Europe				
Algeria	2015	Not eligible	n/a	No
Azerbaijan	2013	Not eligible	n/a	Yes
Iran (Islamic Rep.)^a^	2025	Not eligible	n/a	Yes
Kyrgyzstan	2015	Yes	n/a	Yes
Saudi Arabia	2015	Not eligible	n/a	No
Tajikistan	2015	Yes	n/a	Yes
Turkey	2015	Not eligible	n/a	No
Uzbekistan	2015	Yes	n/a	Yes
The Americas				
Argentina	NNEG	Not eligible	n/a	No
Belize^b^	2020	Not eligible	yes	Yes
Costa Rica^b^	2020	Not eligible	yes	Yes
Dominican Republic^b^	2020	Not eligible	yes	Yes
El Salvador^b^	2020	Yes	yes	Yes
Mexico^b^	2020	Not eligible	n/a	No
Nicaragua^b^	2020	Yes	yes	Yes
Panama^b^	2020	Not eligible	yes	Yes
Paraguay	2015	Yes	n/a	Yes
South-East Asia and Western Pacific				
Bhutan	2018	Yes	n/a	Yes
China	2020	Not eligible	n/a	No
Korea, Dem. Rep.	2025	Yes	n/a	Yes
Malaysia	2020	Not eligible	n/a	No
Philippines	2030	Yes	n/a	Yes
Republic of Korea	2017	Not eligible	n/a	No
Solomon Islands	2035	Yes	n/a	Yes
Sri Lanka	2014	Yes	n/a	Yes
Thailand	2030	Yes	yes	Yes
Vanuatu	2025	Yes	n/a	Yes
Vietnam	2030	Yes	yes	Yes
Sub-Saharan Africa				
Botswana	2018	Yes	yes	Yes
Cape Verde	2020	Yes	n/a	Yes
Namibia	2020	Yes	yes	Yes
Sao Tome and Principe	2020	Yes	n/a	Yes
South Africa	2018	Not eligible	yes	Yes
Swaziland	2015	Yes	yes	Yes

Although these 34 malaria-eliminating countries form the basis of this review, the UCSF Global Health Group’s Malaria Elimination Initiative now identifies 35 malaria-eliminating countries based on progress around the world over the last 5 years [23]

*NNEG* No National Elimination Goal

^a^While not eligible for a new allocation under the NFM, Iran has funding through the Global Fund from a previous 5 year grant signed in 2011

^b^Elimination goal of 2020 declared under the EMMIE regional initiative

As the largest international financier to national malaria programmes, the Global Fund to Fight AIDS, Tuberculosis, and Malaria (GFATM) has played a critical role in reducing global malaria burden. Between 2000 and 2011, global financing for malaria increased 18-fold, largely due to the creation of the GFATM in 2002 [4]. From inception until 2011, the GFATM granted funding through a “round” system whereby countries would submit proposals that were evaluated based on technical soundness, alignment with national strategy, and capacity for implementation [5]. Under this old funding model, a total of US$8.65 billion had been disbursed for malaria, 93 % of which was spent on high burden countries [2]. The remaining 7 % disbursed by the GFATM accounted for the largest source of donor assistance for 19 of the 34 malaria-eliminating countries that received support from the GFATM. Although it is a small percentage of the overall GFATM malaria portfolio, this amount has catalyzed national progress toward elimination [2], helping to reduce malaria cases in the 34 malaria-eliminating countries collectively by 85 % between 2000 and 2013 [6].

In an effort to become more transparent and systematic, the GFATM created the new funding model (NFM) in 2012 to increase value for money and focus investments to hardest hit countries with fewer available financial resources [7]. With the NFM, the GFATM formalized their allocation methodology, largely determined by disease burden and gross national income (GNI) per capita, which emphasized their priority on investments in higher burden, lower income countries [8]. Implemented during the 2014–2016 funding cycle, the NFM offers a pre-calculated allocation to each country for human immunodeficiency virus (HIV), tuberculosis (TB), and malaria.

Under the NFM, countries are first assigned to one of four bands based on their disease burden and income level (Table 2). Then, the allocation formula is applied to determine the country’s national allocation, which includes any unspent money left over from grants under the old funding model, plus a new allocation amount. The malaria disease burden is calculated using the number of deaths + the number of cases + 0.5×incidence + 0.5×mortality rate, based on 2000 malaria incidence data (taken from the World Health Organization), and country income level defined by GNI per capita [9].

**Table 2:** Band assignments for malaria-eliminating countries eligible for GFATM national malaria funding

Band 1 Lower income, high burden	Band 2 Higher income, high burden
Vietnam	Korea, Dem. Rep.
	Kyrgyzstan
	Nicaragua
	Sao Tome and Principe
	Solomon islands
	Tajikistan
	Uzbekistan

Band 3 Lower income, Low burden	Band 4 higher income, low burden
Botswana	Bhutan
Namibia	Cape Verde
Philippines	El Salvador
Swaziland	Paraguay
Thailand	Sri Lanka
	Vanuatu

Source: The Global Fund to Fight AIDS, Tuberculosis and Malaria. *Overview of the Allocation Methodology (2014*–*2016): The Global Fund’s new funding model .*2014 http://www.theglobalfund.org/documents/fundingmodel/FundingModel_OverviewAllocation_Methodology_en/. (12 January 2016, date last accessed)

Once the national allocation is determined and publicly announced, countries can develop a concept note for submission to the GFATM. During concept note development and revisions, the country dialogue process is open and countries can make additional modifications to the allocation. Such adjustments include changes to the disease allocation split between HIV, TB, and malaria or other adjustments based on the willingness to pay criteria, defined by the amount the country is willing to invest in their own programmes beyond the required counterpart financing.

The final concept note is then reviewed by the GFATM’s Grant Approvals Committee. The committee can approve eligible countries for additional incentive funding, defined by the GFATM as “a special reserve of funding available on a competitive basis awarded to applications that demonstrate the greatest potential for high impact with additional funds” [10]. Incentive funding can increase the national allocation up to 15 % and is only available to eligible countries in bands 1–3.

Apart from the national allocations, the GFATM approved regional grants under the NFM to three regions that applied for malaria funding within an amount set aside for regional investments. As of January 2016, three regional grants have been signed: the Elimination 8 (E8) [11] in southern Africa, the Elimination of Malaria in Mesoamerica and the Island of Hispaniola (EMMIE) [12] and the Regional Artemisinin-resistance Initiative (RAI) [13] in the Mekong Region. While national grants tend to focus on in-country commodities and activities, regional grants can play a complementary role, supporting activities that may not be funded through country programmes, such as cross-border surveillance programmes.

Since the GFATM has been such a significant supporter of malaria-eliminating countries, which are by definition, low burden and typically middle-income, and the financial impact of the NFM’s funding methodology is not clear, the authors initiated an analysis to understand the projected increase or decrease national and regional funding from the GFATM to the 34 eliminating countries.

## Methods

### Countries included in this analysis

As of 2010, 34 countries have been identified as malaria-eliminating [1]. Of these, 26 countries were included in the analysis; all met at least one of the following criteria: recently eligible for a GFATM malaria grant under the old funding model; has an active malaria grant from the GFATM; is eligible for a malaria grant under the NFM; and/or is expected to receive funds from the GFATM under a regional malaria grant. The list of countries with their stated national elimination goal is given in Table 1. Eliminating countries that have never been eligible for malaria funding from GFATM or that hold membership to the Group of 20 major economies were excluded from the analysis (Algeria, Argentina, China, Malaysia, Mexico, Republic of Korea, Saudi Arabia, and Turkey).

Eligibility status of the 34 eliminating countries generated by the GFATM are shown in Table 1. Nineteen of the 34 eliminating countries are eligible for NFM national malaria funding with allocation amounts ranging from US$500,000 to US$27 million. Although 19 countries are eligible for national malaria grants and were given allocations in the NFM, four did not receive an allocation with any additional funding apart from the existing, unspent funds from previous grants: Kyrgyzstan, Tajikistan, Thailand, and Vanuatu. Five countries are not eligible for national malaria grants, but are expected to receive funds through a regional malaria grant: Belize, Costa Rica, Dominican Republic, Panama, and South Africa.

### Analysis on national level funding changes

Using publicly available GFATM grant data [14] collated in Microsoft Excel 2010, average annual funding from the old funding model was calculated using the total disbursed amounts from each country’s most recent active malaria grant(s) averaged over the respective grant start date through December 31, 2013, the GFATM specified cut-off date for the round based system. Disbursed amounts rather than the signed amounts in grant agreements from the old funding model were used in order to avoid “double counting” of money not yet disbursed that will later be incorporated into the new NFM national allocation. Using the average disbursements from the entire previous grant(s), rather than the last 3 years under the old funding model, ensures that this analysis compares previous full grants to potential full grants, while capturing any programme scale-up or frontloading.

Estimated NFM average annual allocation amounts were calculated by averaging the GFATM specified national allocation [7] over the 4 year period of 2014–2017. This time period was used since the next GFATM replenishment will take place in the last quarter of 2016. Thus, countries will likely not receive new funding until mid-2017. No regional grant amounts were included in this portion of the analysis.

Average annual grant amounts disbursed under the old funding model were compared to average annual national allocated amounts under the NFM to determine the percent change between old and new average annual funding.

A cumulative percent change between the old funding model and NFM was calculated between the sum total of the old disbursed and new allocated amounts. The cumulative percent change in funding accounts for countries that had an unquantifiable percent change (e.g. those that received no money under the old funding model, and then assigned an allocation under the NFM).

### GFATM NFM regional grants

Funding channeled to malaria-eliminating countries through the E8, EMMIE, and RAI GFATM regional malaria grants was included. While the RAI grant has a predetermined country-level breakdown of funding, in this analysis country shares for EMMIE and E8 were assumed to be divided equally among the countries involved and are described in Table 5.

For eliminating countries included in a regional grant, the country share of regional grant funding was added to the national allocations and a new percent change of funding from the previous funding model compared to the NFM was calculated.

### NFM malaria funding ranges

Since the national malaria allocation is the calculated amount a country is eligible for and not necessarily a final grant amount, the funding range (minimum and maximum) each country could receive was estimated, taking into account potential adjustments and/or additional funding (e.g. regional grant funding under E8, EMMIE, and RAI grants) (Table 3). Because regional grants have already been signed, regional funding amounts remain constant in this portion of the analysis.

**Table 3:** Potential adjustments and additional funding to national allocations

Potential dimension for adjustments	Definition	Adjustment	Timing of adjustment
Willingness to pay	Amount the country is willing to put forth beyond the required counterpart financing. The amount is negotiated between each country and the GFATM	−15 % of national allocation if criteria is not met	During country dialogue
Disease split between HIV, TB, malaria	Amount of funding allocated to each disease, decided upon by the country coordinating mechanism	Up to ± 10 % of the national allocation amount for each disease	During country dialogue
Incentive funding	Aimed to reward high impact, well preforming projects	+15 % for eligible countries (bands 1–3)	During grant-making with the Grant Approvals Committee
Additional Funding			
Regional grant funding	Any funding granted to a country from a regional grant (E8, EMMIE, and RAI)—this amount would be additive to any national grants	Country share breakdown per regional grant amounts	Independent of national grant process

Source: global fund to fight AIDS, tuberculosis and malaria resource book for applicants: The global fund’s new funding model (2014)

In order to access the full national allocation, each country must meet a conditional counterpart financing requirement, or a minimum level of government contribution to the national disease programme as a share of total government financing plus GFATM financing for that disease [9]. The counterpart financing requirement is based on a sliding scale of income level: low-income countries must reach a minimum threshold contribution of 5 %, lower LMICs must reach a minimum threshold contribution of 20 %, upper LMICs must reach a minimum threshold contribution of 40 %, and upper–middle-income countries must reach a minimum threshold contribution of 60 %. Countries must then meet their willingness to pay criteria, which is an additional amount beyond the counterpart financing requirement. If a country does not meet their willingness to pay criteria, 15 % of the national allocation for each disease component can be withheld. Furthermore, during the country dialogue process, the country-level stakeholder partnership that manages the proposals and grants, also known as the Country coordinating mechanism, can adjust the GFATM’s suggested national disease split, potentially transferring up to 10 % of malaria funding to supplement HIV or TB or vice versa. Table 3 summarizes potential adjustments and additional funding used to determine the range of a country’s allocation from the GFATM.

Percentage adjustments were calculated from the suggested national allocation amounts announced by the GFATM in March 2014 [15]. To calculate the minimum funding for a country’s malaria programme, the national allocations were decreased by 15 % to simulate unmet willingness to pay criteria and by an additional 10 % to account for a possible Country coordinating mechanism decision to move malaria funding to another disease. Independent of national allocation adjustments, any country’s share of regional grants is consistent in the minimum funding amounts.

The maximum potential funding was then calculated based on meeting the willingness to pay criteria, a 10 % disease split increase, a 15 % increase for incentive funding (for those in bands 1–3 that are eligible), and additional regional grant amounts.

### NFM minimum and maximum funding amounts compared to the old funding model

Both the minimum and maximum funding amounts (national allocations plus regional grants) were averaged over the 4-year period of 2014–2017 and compared to the average annual disbursements under the old funding model to determine the range of percent change in funding for eligible eliminating countries.

## Results

### Funding changes to the GFATM’s malaria portfolio

**Fig. 1:**
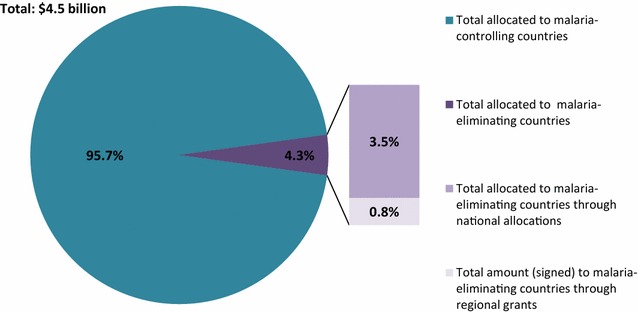
The GFATM malaria portfolio under the new funding model including national allocations and signed regional malaria grants. The majority (95.7 %) of the Global Fund’s portfolio for malaria under the new funding model is allocated to go to countries working to control malaria and 4.3 % is allocated to malaria-eliminating countries

Under the NFM, 4.3 % of the GFATM’s malaria portfolio of US$4.5 billion (including national allocations and regional malaria grant funding) is allocated to the focus countries in this paper (Fig. 1). Of the 4.3, 0.8 % of the malaria portfolio supports eliminating countries through three regional grants for malaria: E8, EMMIE, and RAI. Under the NFM, the total portion of the malaria portfolio going to malaria-eliminating countries is lower (4.3 %) than under the old funding model (7 %).

### Analysis on national level funding changes

Changes in annual national funding between the most recent grant(s) under the old funding model and the average annual allocation under the NFM are shown in Table 4. Overall, there is a projected 31 % decrease in average annual funding during the 2014–2017 timeframe for malaria-eliminating countries. Twelve countries (Azerbaijan, Cape Verde, Dominican Republic, Iran, Kyrgyzstan, Philippines, Solomon Islands, Sri Lanka, Tajikistan, Thailand, Uzbekistan and Vanuatu) are expected to see an extreme decrease (30–100 %) in funding, with three (Democratic People’s Republic of Korea, Swaziland and Vietnam) expected to have a less severe decrease in funding (1–29 %). Four countries (Bhutan, Namibia, Nicaragua, and São Tomé and Príncipe) will see increases in funding, ranging between 1 and 54 %. The percent change for three countries (Botswana, El Salvador, and Paraguay) could not be quantified, as they have not received any prior funding from the GFATM, but allocations and potential grants to these countries would be an increase. The remaining four countries (Belize, Costa Rica, Panama, and South Africa) have no change in national funding.

**Table 4:** Average annual disbursements under the old funding model versus average annual NFM national allocations 2014–2017

Countries	Average annual disbursements before the NFM as of dec 31st, 2013^b^	Average annual allocation under NFM: 2014–2017	Percent change^a^
Eastern Mediterranean and Europe			
Azerbaijan	$1,049,387	$0	−100 %
Iran	$5,461,418	$0	−100 %
Kyrgyzstan	$884,028	$113,074	−87 %
Tajikistan	$2,721,312	$335,802	−88 %
Uzbekistan	$578,319	$350,280	−39 %
Regional subtotal	*$10,694,464*	*$799,156*	*−93* %
The Americas			
Belize	$0	$0	0 %
Costa Rica	$0	$0	0 %
Dominican Republic	$1,592,747	$0	−100 %
El Salvador	$0	$963,783	+
Nicaragua	$2,431,682	$2,921,343	20 %
Panama	$0	$0	0 %
Paraguay	$0	$1,338,783	+
Regional subtotal	*$4,024,429*	*$5,223,908*	*30* *%*
South-East Asia and Western Pacific			
Bhutan	$595,598	$641,075	8 %
Korea, Dem. Rep.	$4,878,128	$3,966,350	−19 %
Philippines	$8,594,847	$5,543,637	−36 %
Solomon Islands^c^	$2,329,166	$1,617,630	−31 %
Sri Lanka	$5,310,434	$3,194,798	−40 %
Thailand	$13,611,345	$8,914,463	−35 %
Vanuatu^c^	$1,552,777	$813,042	−48 %
Vietnam	$4,895,794	$3,778,554	−23 %
Regional subtotal	*$41,768,089*	*$28,469,547*	*−32* *%*
Sub-Saharan Africa			
Botswana	$0	$1,282,149	+
Cape Verde	$633,015	$320,537	−49 %
Namibia	$2,431,682	$3,018,565	24 %
Sao Tome and Principe	$1,807,650	$2,733,377	51 %
South Africa	$0	$0	0 %
Swaziland	$1,420,225	$1,290,603	−9 %
Regional subtotal	*$6,292,571*	*$8,645,232*	*37* *%*
Total	*$62,779,553*	*$43,137,843*	*−31* *%*

^a^ + indicates a percent change was unquantifiable (e.g. a country who had received no previous GFATM funding is allocated funding under the NFM.)

^b^This is calculated by taking the total grant disbursement through 2013 and dividing it by each grant’s start date through 31-December-2013

^c^These countries compose the multi-country Western Pacific, whose previous grant was split 60/40 (Solomon Islands:Vanuatu)

When percent changes for the national allocations were aggregated regionally (also shown in Table 4), it is clear that the Eastern Mediterranean and Europe and the South-East Asia and Western Pacific regions are the hardest hit with declines of 93 and 32 %, respectively. The majority of the eliminating countries in these regions are projected to experience mild to steep declines in funding. Malaria-eliminating countries in the Americas are expected to see an overall increase of 30 %, while malaria-eliminating countries in sub-Saharan Africa will likely have an overall 37 % increase in allocations under the NFM.

### GFATM NFM regional grants

Regional grants provide US$39.6 million over 3 years in extra support for 12 malaria-eliminating countries located in southern Africa, Central America, and the Mekong region (as shown in Table 5) and boost overall funding for malaria elimination from −31 % to an increase of 32 %. Adding regional grant country shares to national funding have a clear positive affect to funding. With the addition of regional funding, malaria-eliminating countries in the Americas are expected to see a cumulative 171 % increase in funding compared to the old funding model. Similarly, malaria-eliminating countries in South-East Asia and Western Pacific are expected to see an overall 28 % increase in funding, and malaria-eliminating countries in sub-Saharan Africa are expected to see an overall 179 % increase in funding. No regional grant funding for malaria has been provided to malaria-eliminating countries in the Eastern Mediterranean and European regions.

**Table 5:** Regional Grants for malaria under the NFM

GFATM regional grant for malaria	Total grant amount	Total estimated to malaria-eliminating countries included in grant scope	Malaria-eliminating countries included in regional grant scope
Elimination 8 (E8)	$17,800,000	$8,900,000	Botswana, Namibia, Swaziland, South Africa
Elimination of Malaria in Mesoamerica and the Island of Hispaniola (EMMIE)	$10,000,000	$5,666,668	Belize, Costa Rica, Dominican Republic, El Salvador, Nicaragua, and Panama
Regional Artemisinin-resistance Initiative (RAI)	$100,000,000	$25,000,000	Thailand and Vietnam

The E8 is not structured such that it has country specific breakdowns of funding. For this analysis, it was assumed that the US$17.8 million is divided equally among the eight countries (Angola, Botswana, Mozambique, Namibia, South Africa, Swaziland, Zambia and Zimbabwe)

The US$10 million EMMIE regional grant covers ten countries, five of which are eligible for startup funding (Costa Rica, Belize, El Salvador, Mexico, Panama), and nine of which are eligible for payouts (all but Mexico). EMMIE is a cash-on-delivery model and of the US$10 million, US$3 million will go to Population Services International as the Principal Recipient. Because it will not be known which countries will be successful in meeting targets until the end of years 2 and 3, this analysis assumed that the remaining amount (US$7 million) was evenly split over the nine eligible countries and added to startup funding, if applicable

Fifteen percent of the US$100 million RAI regional grant goes to Vietnam and 10 % goes to Thailand

### NFM malaria funding ranges

**Fig. 2:**
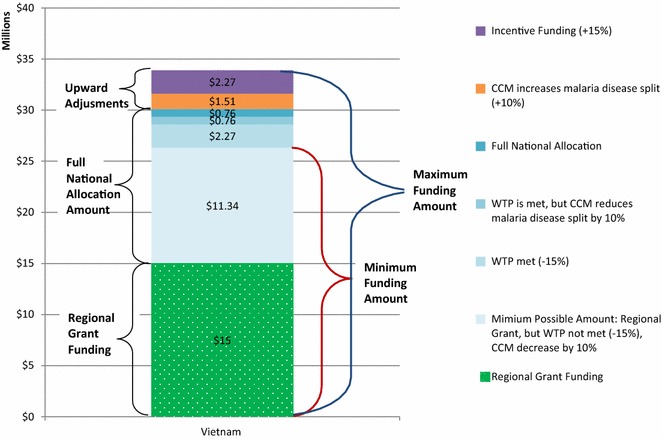
Estimated global fund NFM malaria funding range for Vietnam as an example, for the period of 2014–2017 using adjustments and additional funding. Depending on various factors, funding for Vietnam can range 25 % more or 25 % less than their stated allocation amount. *CCM* country coordinating mechanism. *WTP* willingness to pay

As an example, Fig. 2 illustrates the breakdown of the estimated funding range available for Vietnam for the period of 2014–2017. The range is determined by the adjustments made during the country dialogue process and the addition of regional grant funding. The area at the bottom of the funding range represents Vietnam’s portion (US$15 million) of the RAI regional grant. The solid fill area represents the full national allocation, which totals US$15 million, with the various shaded areas showing the portion of the national allocation Vietnam would receive based on unmet willingness to pay criteria and/or a reduction of the disease split amount. Possible upward adjustments include an increase in disease split funding (an additional US$1.51 million) and successful award of incentive funding (US$2.27 million) and are represented at the top of the funding range. Accordingly, Vietnam’s minimum possible funding of about US$26 million would include the RAI regional grant share plus the minimum national allocation (unmet willingness to pay and a Country Coordinating Mechanism decision to move 10 % of malaria funding to HIV or TB). Vietnam’s maximum funding amount of nearly US$34 million includes the RAI regional grant share plus the full national allocation and all upward adjustments (a Country coordinating mechanism decision to increase malaria by 10 % and successful award of incentive funding).

**Fig. 3:**
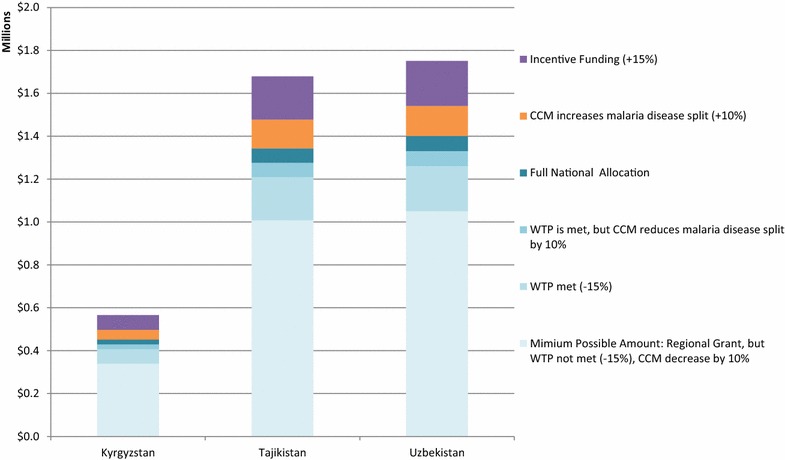
Estimated global fund NFM malaria funding ranges for malaria-eliminating countries in the Eastern Mediterranean and Europe regions, for the period of 2014–2017 using adjustments and additional funding. Minimum funding under the NFM would be calculated as: Full national allocation—10 % of national allocation approved to be reallocated to AIDS or TB from malaria—15 % of national allocation for unmet willingness to pay criteria+ any regional grant funding. Maximum funding would include: Full national allocation+ 10 % of national allocation for additional disease resources decided by the country coordinating mechanism+ 15 % incentive, if eligible, + any regional grant funding. Azerbaijan and Iran do not have a NFM allocation and no countries in this region have been granted funding through a regional grant. *CCM* country coordinating mechanism. *WTP* willingness to pay

**Fig. 4:**
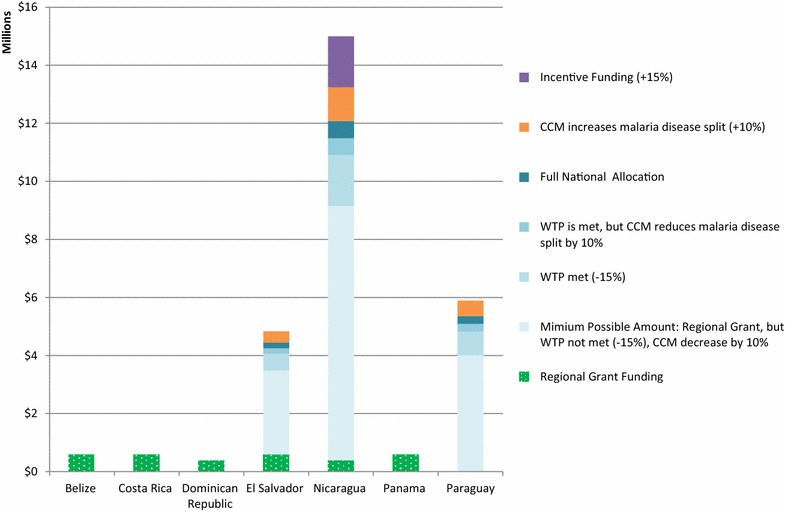
Estimated global fund NFM malaria funding ranges for malaria-eliminating countries in the Americas, for the period of 2014–2017 using adjustments and additional funding. Minimum funding under the NFM would be calculated as: Full national allocation—10 % of national allocation approved to be reallocated to AIDS or TB from malaria—15 % of national allocation for unmet willingness to pay criteria + any regional grant funding. Maximum funding would include: Full national allocation + 10 % of national allocation for additional disease resources decided by the country coordinating mechanism + 15 % incentive, if eligible, + any regional grant funding. Belize, Costa Rica, Dominican Republic, and Panama did not get a new allocation amount from the GFATM, but will receive funding through the EMMIE regional grant if they meet the targets agreed upon in the grant. *CCM* country coordinating mechanism. *WTP* willingness to pay

**Fig. 5:**
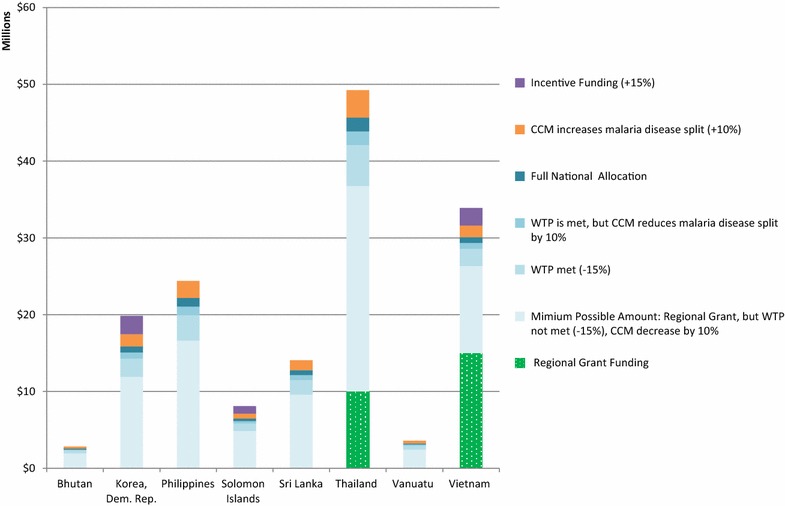
Estimated global fund NFM malaria funding ranges for malaria-eliminating countries in the South-East Asia and Western Pacific, for the period of 2014–2017 using adjustments and additional funding. Minimum funding under the NFM would be calculated as: Full national allocation— 10 % of national allocation approved to be reallocated to AIDS or TB from malaria—15 % of national allocation for unmet willingness to pay criteria + any regional grant funding. Maximum funding would include: Full national allocation + 10 % of national allocation for additional disease resources decided by the Country Coordinating Mechanism + 15 % incentive, if eligible, + any regional grant funding. *CCM* country coordinating mechanism. *WTP* willingness to pay

**Fig. 6:**
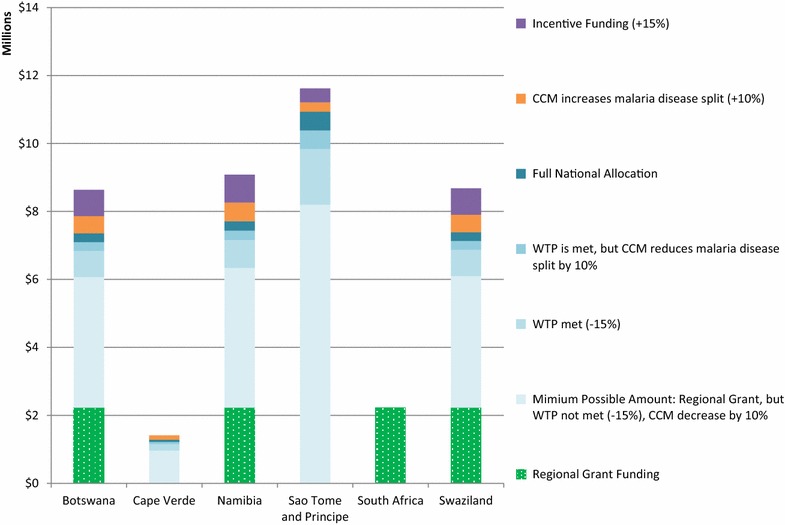
Estimated global fund NFM malaria funding ranges for malaria-eliminating countries in sub-Saharan Africa for the period of 2014–2017, using adjustments and additional funding. Minimum funding under the NFM would be calculated as: Full national allocation— 10 % of national allocation approved to be reallocated to AIDS or TB from malaria—15 % of national allocation for unmet willingness to pay criteria + any regional grant funding. Maximum funding would include: Full national allocation + 10 % of national allocation for additional disease resources decided by the country coordinating mechanism + 15 % incentive, if eligible, + any regional grant funding. *CCM* country coordinating mechanism. *WTP* willingness to pay

Applying the same structure, Figs. 3, 4, 5, and 6 show the possible funding ranges for eligible eliminating countries for the period of 2014–2017, by region. The possible adjustments and additional regional grant funding have the potential to change the allocations by either 25 % more or less than the amount originally communicated to the countries in March 2014. In the Americas, Belize, Costa Rica, Dominican Republic and Panama are not eligible for national grants and thus do not have national allocations, however they can receive funding through the regional EMMIE award. Similarly, South Africa is not eligible for a national allocation, however is assumed to receive one-eighth of the E8 regional grant.

### NFM minimum and maximum funding amounts compared to the old funding model

**Fig. 7:**
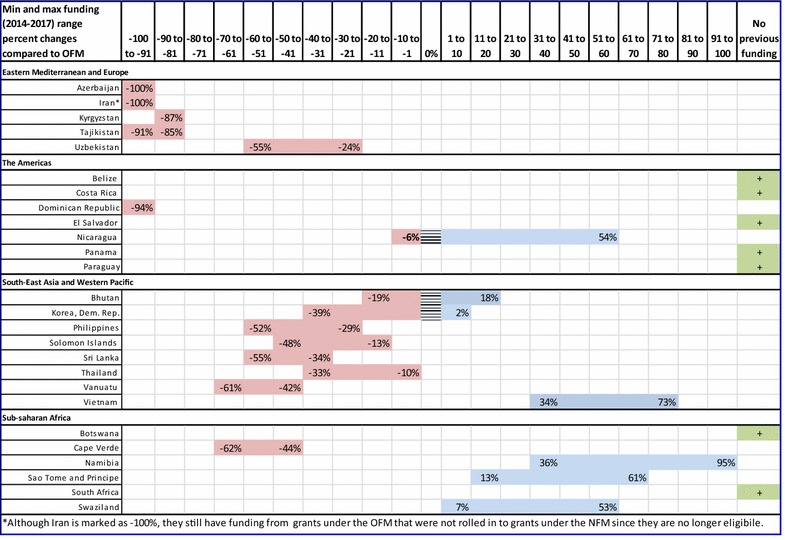
Percent changes between the average annual disbursements under old funding model to average annual NFM minimum and maximum funding amounts. If the minimum and/or maximum percentage falls between −100 and −1, then the NFM minimum and/or maximum amount would be less than the country would have received under the old funding model. Conversely, if the minimum and/or maximum percentage falls between 1 and 100, the NFM minimum and/or maximum amount would be more than the country would have received under the old funding model. Countries marked with a “+” have an unquantifiable percent change in funding (e.g. a country who had received no previous GFATM funding is now able to receive funding)

The range of percent differences between the estimated minimum and maximum average annual allocations for 2014–2017 determined in Figs. 3, 4, 5, 6 are compared to average annual disbursements under the old funding model and are shown in Fig. 7. Percentages on the left side of a country’s range indicate the percent change between a country’s minimum funding amount compared to their funding under the old funding model. Similarly, percentages to the right side of the range indicate the change between a country’s maximum funding amount compared to funding under the old funding model. In the best case scenario (receiving maximum funding from the GFATM for malaria), 46 % of the countries included in this analysis will still see decreases in funding (Cape Verde, Philippines, Solomon Islands, Sri Lanka, Tajikistan, Thailand, Uzbekistan, and Vanuatu). For countries like Bhutan, Namibia, Nicaragua, Sao Tome and Principe, Swaziland and Vietnam, the extra adjustments, if made, could mean a considerable increase in support for their elimination efforts. Azerbaijan, Dominican Republic, Iran, and Kyrgyzstan are no longer eligible for funding due to either their low malaria burden or income level.

## Discussion

Under the NFM, a total of US$4.5 billion has been allocated to 75 countries deemed eligible for GFATM malaria support through national allocations and countries included in three regional grants to E8, EMMIE, and RAI [7]. The proportion of the overall GFATM malaria portfolio to eligible malaria-eliminating countries has decreased—from 7 % under the old funding model to 4.3 % under the NFM, less than a quarter of which is from funding through the three regional grants. Despite this small and shrinking portion of GFATM funding, this money has been and will continue to be catalytic in accelerating toward malaria elimination in these countries. In contrast, roughly 20 % (US$0.9 billion) of the GFATM malaria portfolio goes to just two countries (Democratic Republic of Congo and Nigeria) [7]. Thirty percent (US$1.3 billion) goes to ten countries (Burkina Faso, Cameroon, Côte d’Ivoire, Ethiopia, Ghana, Kenya, Mozambique, Tanzania, Sudan and Uganda) [7].

Currently, there is a projected overall decrease of 31 % in allocated national funding to eliminating countries from the GFATM. The change in total allocations to the eligible eliminating countries compared to previous disbursements under the old funding model varies widely by country: some countries are allocated up to 100 % more than previous disbursements and other countries are allocated significantly less. However, this allocation formula provides a preliminary guideline for the signed grant amounts, which are shaped by the Country coordinating mechanisms who have the opportunity to negotiate for additional resources based on the country’s needs and timelines. This flexibility in the NFM allows for countries to take full ownership of the grants once implemented on the ground.

Still, uncertainties remain for countries around the grant making process and the adjustments that could be applied, including the domestic counterpart financing requirement and willingness to pay criteria. All allocations are conditional on countries reaching their minimum counterpart financing requirement, based on income level. While 78 % of financing for malaria elimination is generated at the domestic level, many of the low-income and LMICs depend heavily upon GFATM financing (such as Bhutan, Nicaragua, Philippines, the Solomon Islands, Sri Lanka, and Vietnam) [16] and any reduction in donor financing could hinder their efforts to eliminate malaria and prevent re-introduction. Past estimates calculated from World Malaria Report 2012 data for years 2005 through 2010 indicate that roughly 20 % of eliminating countries have not historically met what would be a 5–60 % domestic counterpart financing requirement [4].

Along with the counterpart financing requirement, the willingness to pay adjustment is an effort to increase domestic financing and promote sustainability of GFATM investments. While intended to support sustainability, the domestic funding contribution criteria require additional facilitation from the GFATM, especially for countries transitioning to higher income levels. The GFATM can help countries advocate for increased domestic financing through a variety of channels, using tools such as the WHO’s Global Technical Strategy for Malaria 2016–2030 [17] and Roll Back Malaria’s Action and Investment to Defeat Malaria 2016–2030 [18] to demonstrate the strategies and economic investment cases for funding, and by leveraging regional organizations such as the African Leaders Malaria Alliance [19], the Asia Pacific Leaders Malaria Alliance [20], and the Asia Pacific Malaria Elimination Network [21] to help garner the high-level political support and to implement tools needed to increase domestic financing.

Analysis of the funding ranges suggests that projected funding amounts are quite variable. Countries could receive roughly 25 % more or 25 % less than their allocated amounts, as exemplified by the variance in Vietnam’s funding range for the period of 2014–2017. If Vietnam does not meet the willingness to pay requirement and their Country Coordinating Mechanism prioritizes HIV or TB over malaria, their GFATM’s national malaria allocation can decrease from about US$15 million to just over US$11 million (about 25 % less than the full national allocation amount). In this case, the minimum funding amount would equal a US$11 million national allocation plus US$15 million in regional grant funding. Furthermore, if Vietnam’s minimum funding amount is compared to their average funding under the old funding model, they are expected to see a 34 % increase in funding. If the Country Coordinating Mechanism prioritizes malaria funding, and the GFATM determines the country should receive their full incentive allocation in addition to their national allocation and regional grant funding, it is possible that Vietnam could receive almost US$34 million (about 73 %) more funding than under the old funding model. However, this is not the case for about half of the malaria-eliminating countries. Even if they receive their maximum funding amount, 46 % of eliminating countries are projected to see a decrease in funding from the GFATM under the NFM when compared to the old funding model. It is unlikely that many countries would receive the estimated maximum funding calculated by the post-allocation adjustments.

These findings suggest an unpredictable environment for malaria programmes to operate in. Due to competing disease priorities, some eliminating countries may not be able to continue to adequately fund national malaria programmes, putting them at higher risk of resurgence. Historical evidence suggests that if malaria funds are interrupted, programmes are weakened, or interventions are disrupted before malaria has been eliminated, there is a danger of malaria resurgence [22]. Furthermore, this reduction in funding is not limited to malaria-eliminating countries; many control countries such as Ethiopia, Haiti, Côte d’Ivoire and Uganda are also projected to see a decline in funding [7], straining resources in these settings as well. To mitigate the risk of resurgence, account for progress in burden reduction, and address the malariogenic potential of endemic countries, the GFATM has used malaria epidemiology data from the World Health Organization from 2000 to 2010 in the allocation methodology.

With the addition of regional grants, a 31 % decrease in national funding is augmented to a cumulative 32 % increase in funding for malaria-eliminating countries. Regional trend analysis suggesst the malaria-eliminating countries in the Eastern Mediterranean and Europe region are expected to see a 93 % decrease in GFATM national financing, mainly due to steep declines in malaria cases. Malaria-eliminating countries in South-East Asia and Western Pacific are expected to experience an overall 32 % decline in aggregated national funding, as countries such as the Philippines, the Solomon Islands, Sri Lanka, and Vanuatu all are expected to experience decreases in funding ranging from 30 to 50 %. However, with the addition of the RAI regional grant, the eliminating countries in the region are expected to see a 28 % increase in funding, mainly through RAI support to Thailand and Vietnam. The RAI grant is a particularly strategic investment and is expected to have a positive impact for elimination in the region, providing additional support to higher burden Mekong countries. This is especially critical given the serious threat of anti-malarial drug resistant malaria. Despite the Dominican Republic’s recent ineligibility for malaria funding, eliminating countries in the Americas are expected to see an overall 171 % increase with the additional funding through EMMIE, particularly to countries that would otherwise be ineligible for national malaria funding. The malaria-eliminating countries in sub-Saharan Africa are expected to see an overall 179 % increase in funding due to the addition of the E8 grant funds and because Botswana, although previously eligible, did not receive funding under the old funding model but did receive a malaria allocation of roughly US$1.3 million under the NFM. The E8 regional grant, which will support eight countries in the southern Africa region, also includes South Africa, who is otherwise ineligible for national malaria funding.

Despite providing much needed additional funding for elimination, funds granted through regional channels will likely not fill all the gaps from reduced national level allocations as they usually will not cover country specific activities or necessary commodity procurement. Regional grants can, however, leverage country-level efforts by providing complementary investments to support cross-border initiatives and collaboration that would not otherwise be included in country grants. Another benefit is that the regional approach is two-pronged; it supports both high- and low- transmission countries by creating a platform for data and information sharing and provides an opportunity for enhanced collaboration between countries.

Because the eliminating countries are a critical part of a global movement toward eradication and maintaining essential national level funding is crucial, a mix of regional and country investments by the GFATM can leverage the gains already made toward eradicating malaria. Country grants support core malaria interventions, while regional grants support collaborative surveillance platforms and demonstrate strong value for money by driving economies of scale among low burden countries. The regional grants can also hold regions accountable for reaching goals for elimination and eventual global eradication by jointly monitoring national and regional activities that are mutually reinforcing. Funding from the GFATM has been essential to many of the eliminating countries, and maintaining this level of funding, through a mix of national and regional funding streams, will be needed in order protect investments and sustain progress toward a malaria-free world.

## Limitations

The adjustments made to the national allocation introduce important limitations in this analysis, which affect the quantification of the funding ranges for each country. These ranges were quantified based on the information provided by the GFATM; however, other factors are evaluated on a case-by-case basis and how decisions affect funding is ultimately determined by the GFATM and the Country coordinating mechanism. Thus, these funding ranges should be taken as estimations to provide guidance on potential funding ranges from the GFATM.

Another major limitation is the analysis is that due to a significant time lag between programme implementation and impact on malaria epidemiology, the analysis cannot fully assess the financial impact on in-country malaria burden.

There are likely other benefits of the NFM on malaria-eliminating countries that are outside the scope of this analysis. GFATM funding for health system strengthening, separate from the three disease streams, would likely improve overall outcomes across the board.

## Conclusion

Funding from the GFATM has been critical for many countries to accelerate progress toward malaria elimination. As the GFATM prioritizes higher burden, lower income countries, national funding streams to many eliminating countries are projected to be at risk. A decrease in national funding could reverse all the hard-earned gains and returns on the GFATM’s investment to-date. For some of these eliminating countries, regional grants for malaria have augmented funding for elimination activities and helped encouraged regional collaboration but they are unable to fill all the gaps in funding created through reductions in national funding. Without strong national malaria programmes, regional grants may be less effective in achieving regional goals. By creating a more nuanced allocation formula or a mix of other mechanisms to invest in malaria eliminating countries, the GFATM has an opportunity to ensure their previous investments in malaria are not lost. As the global community sets its sights on a malaria-free world, the GFATM’s continued investments in both high and low-burden countries will signal alignment with countries and regions that are paving the way toward malaria elimination and eventual eradication.

## Availability of supporting data

Data supporting the results of this study are available from http://www.theglobalfund.org/.

## Abbreviations

GFATM: Global Fund to Fight AIDS, Tuberculosis, and Malaria
GNI: gross national income
HIV: human immunodeficiency virus
E8: Elimination 8
EMMIE: Elimination of Malaria in Mesoamerica and the Island of Hispaniola
LMICs: lower-middle-income countries
NFM: new funding model
RAI: Regional Artemisinin-resistance Initiative
TB: tuberculosis
